# *xINTERPDF*: a graphical user interface for analyzing intermolecular pair distribution functions of organic compounds from X-ray total scattering data

**DOI:** 10.1107/S1600576718012359

**Published:** 2018-09-20

**Authors:** Chenyang Shi

**Affiliations:** a Drug Product Development, AbbVie Inc., 1 North Waukegan Road, North Chicago, IL 60064, USA

**Keywords:** X-ray total scattering, pair distribution function, Python programming, graphical user interfaces

## Abstract

A new software program, *xINTERPDF*, that analyzes the intermolecular correlations in organic compounds *via* measured X-ray total scattering data is described.

## 1. The crystallographic problem   

Structures of organic compounds are more complex than their inorganic counterparts, which have usually a network structure, representing a giant ‘molecule’. Organics, on the other hand, have strong intramolecular bonds but much weaker intermolecular interactions, making them prone to structural disorder. Another complexity comes from the weak X-ray scattering of light elements (C, H, O, N *etc.*) which are the building blocks of organic compounds. The atomic pair distribution function (PDF) calculated from X-ray (and/or neutron, electron) total scattering has been demonstrated to be a valuable tool for investigating structures of disordered and amorphous organic compounds (Shi *et al.*, 2017 ▸; Prill *et al.*, 2015 ▸, 2016 ▸; Benmore & Weber, 2011 ▸; Rademacher *et al.*, 2012 ▸; Chen *et al.*, 2014 ▸; Gorelik *et al.*, 2015 ▸). Although existing tools such as *DiffPy-CMI* (Juhás *et al.*, 2015 ▸) and *XISF* (Mou *et al.*, 2015 ▸) can be used for this problem, a new software program that provides a user-friendly graphical user interface (GUI, as opposed to script-based programming in *DiffPy-CMI*) and analyzes the data in real space (as opposed to in reciprocal space in *XISF*) is still of great value. This article describes such a program, *xINTERPDF*.

## 2. Method of solution   

In *xINTERPDF*, user-friendly GUIs have been built to facilitate user interactions with the data. It currently supports the following:

(1) The study of intermolecular interaction (*e.g.* hydrogen bonds) by subtracting out the scattering signal of a single molecule in real space.

(2) The PDF model fit of the crystalline organic compound using the method proposed by Prill *et al.* (2015 ▸).

(3) The phase quantification of physical mixtures of organics.

(4) The generation of score/scree plots based on principle component analysis (PCA).

The program is written in the open-source Python programming language (https://www.python.org/) and is distributed to various operating systems using the *Conda* package manager (https://conda.io/docs/).

## 3. Software and hardware environment   

The program runs on both 64-bit Linux and macOS machines. It is written in Python 2.7, using its default *Tkinter* module (https://docs.python.org/2/library/tkinter.html) to create the GUI, *Matplotlib* (https://matplotlib.org/) for visualization, and *NumPy* (http://www.numpy.org/) and *SciPy* (https://www.scipy.org/) for scientific calculations. The sklearn.decomposition.PCA module from *Scikit-Learn* (Pedregosa *et al.*, 2011 ▸) is called for application of PCA. The *DiffPy-CMI* package (Juhás *et al.*, 2015 ▸) is used as a backend for the simulations of PDFs.

## 4. Program specification   

*xINTERPDF* runs in the same way on Linux and macOS systems. The look and feel of the GUI may slightly vary. When studying intermolecular interactions in organics, the simulation of the PDF in real space is finished almost instantaneously. However, it takes a relatively longer time for simulating the PDF of a crystal using the Debye scattering equation (Debye, 1915 ▸). For a typical model fit of a crystalline PDF (*e.g.* d-mannitol with 104 atoms in the expanded cell) in an *r* range up to 40 Å, it takes about ∼10 min to complete on macOS 10.10.3 with a 3.1 GHz Intel Core i7 and 16 GB memory. The usages of phase quantification and PCA return results in real time.

## 5. Documentation and availability   

The home page for the *xINTERPDF* program is https://www.diffpy.org/products/xinterpdf.html, where users may find instructions for installation and the manual for applications. The source code is hosted at GitHub page https://github.com/curieshicy/xINTERPDF.

## 6. Disclosure   

C. Shi is the employee of AbbVie and may own AbbVie stock. The design, study conduct and financial support for this research were provided by AbbVie. AbbVie participated in the interpretation of data, review and approval of the publication.
